# Quality of Life of People Living With Dementia Residing in Nursing Homes: Secondary Analysis of Observational Data

**DOI:** 10.2196/91968

**Published:** 2026-09-15

**Authors:** Dirk Steijger, Mark C Scheper, Robert Frans van der Willigen, Hannah Christie, Marjolein E de Vugt, Hilde Verbeek, Sil Aarts

**Affiliations:** 1 Alzheimer Centrum Limburg, Faculty of Health, Medicine and Life Sciences, Maastricht University, Doctor Tanslaan, 12, Maastricht, Limburg, 6200 MD, The Netherlands, 31 433882222; 2 Department of Health Service Research, CAPHRI Care and Public Health Research Institute, Faculty of Health Medicine and Life Science, Maastricht University, Maastricht, Limburg, The Netherlands; 3 The Living Lab in Ageing & Long-Term Care, Maastricht, The Netherlands; 4 Institute for Communication, Media and Information Technology, Rotterdam University of Applied Sciences, Rotterdam, The Netherlands; 5 Data Supported Healthcare, Research Center Innovations in Care, Rotterdam University of Applied Sciences, Rotterdam, The Netherlands; 6 Faculty of Medicine and Health Sciences, Macquarie University Sydney, Sydney, Australia; 7 Medical Delta Livinglab: Data Supported Healthcare & Innovation, Delft, The Netherlands; 8 School of Population Health, Royal College of Surgeons in Ireland, Dublin, Ireland

**Keywords:** dementia, quality of life, AI, large language model, long-term care, unstructured data

## Abstract

**Background:**

Quality of life (QoL) plays a crucial role in dementia care; however, QoL and its dynamic, context-dependent nature can be difficult to capture among people living with dementia due to challenges in memory and communication, and limitations of self-reported QoL instruments. Observational tools such as the Maastricht Electronic Daily Life Observation (MEDLO) provide narrative descriptions of the daily life of people living with dementia in nursing homes. However, the MEDLO tool was not developed to assess QoL specifically, and it remains unclear to what extent its narrative descriptions reflect aspects of QoL. Analyzing these narrative descriptions is labor-intensive and time-consuming. Recent advances in natural language processing, including large language models (LLMs), offer the potential to analyze these narrative descriptions at scale.

**Objective:**

The study aims to explore whether an LLM can be used to structure existing MEDLO narrative data into interpretable QoL-relevant patterns in people living with dementia. Specifically, this study examines whether N-gram analysis, sentiment analysis, and LLM-based topic modeling can identify recurring language patterns, emotional tone, and semantic clusters that can be mapped to Lawton QoL domains.

**Methods:**

This study conducted a secondary analysis of existing MEDLO observational data from 151 people living with dementia residing in Dutch long-term care. Narrative data had been documented by trained observers, describing activities, interactions, settings, and emotional expressions. For analysis, a local secure pipeline was developed in which GPT-4o-mini was deployed. The pipeline comprised three analytical steps: (1) N-gram frequency analysis, (2) sentiment analysis, and (3) topic modeling. Prompts were iteratively refined through prompt engineering. Coauthors and domain experts reviewed outputs for coherence, contextual plausibility, and relevance to long-term care practice.

**Results:**

A total of 5622 narratives (50,106 words) from 151 people living with dementia were analyzed. The narratives were short, averaging 10.5 (SD 5.80) words per narrative. N-gram frequency analysis identified the frequent documentation of passive activities (*sits at the table*) in limited indoor settings (*living room*). Emotional well-being was often described in positive terms (*smiles* and *laughs*), whereas explicitly negative expressions (*cries* and *distress*) occurred less frequently. Weighted sentiment analysis showed that, although fewer in number, negative expressions carried a stronger intensity, resulting in an overall predominance of negative sentiment across all QoL domains. Topic modeling identified 8 coherent clusters, most of which mapped onto multiple QoL domains, underscoring QoL’s multidimensionality.

**Conclusions:**

LLM-based analyses identified predominantly passive activities with little variation in indoor settings, while people living with dementia were often described as having positive affect. This exploratory study suggests that LLM-based analyses may help structure observational narratives into QoL-relevant patterns, but further validation is needed before such outputs can inform person-centered care practice.

## Introduction

Dementia is a neurocognitive disorder with cognitive decline, leading to impairments in cognitive, social, and behavioral functioning [1,2]. While the symptoms of dementia can negatively affect quality of life (QoL), factors such as individual needs, lived experiences, and environmental support also play a crucial role [3-5]. Lawton conceptualizes QoL in people living with dementia as a multidimensional concept, consisting of 4 domains: behavioral competence (physical health and functional ability), psychological well-being (mood), objective environment (environmental factors), and perceived QoL (the individual’s own evaluation of their functioning, mood, and overall life) [3,6]. This holistic perspective emphasizes that QoL extends beyond symptom severity, with consideration of the way individuals interact with their environment and preserve a sense of identity and autonomy. Improved QoL in people living with dementia has been linked to reduced agitation and depression and to an improved sense of autonomy and satisfaction with care [4,7-10].

As dementia progresses, declines in communication, memory, and executive functions often make it difficult for people living with dementia to recall and express their preferences, emotions, and sources of discomfort. This can reduce the usefulness of self-report QoL measures [11,12]. Other measures include proxy-based questionnaires and systematic observational methods. However, these methods are not always capable of reflecting the dynamics and nuances associated with day-to-day QoL [13,14]. In recent years, observation tools such as the Maastricht Electronic Daily Life Observation (MEDLO) have introduced a more comprehensive assessment approach by integrating quantitative information with narrative descriptions of the behavior, mood, and environment of people living with dementia in nursing homes [15]. These narrative descriptions, hereafter referred to as narrative data, are collected by a trained observer and contain written information about the behavior of an individual and the environmental contextual information in which this behavior occurred. They capture detailed, real-time descriptions of the daily lives of people living with dementia in long-term care settings, offering a unique window into the everyday QoL. However, the MEDLO tool was not developed to assess QoL specifically, as it does not include a predefined QoL scoring system, validated QoL subscales, or direct self-reported evaluations of QoL by the person living with dementia. Therefore, the narrative data may contain signals that are relevant to QoL, but these signals remain implicit. Moreover, manual analysis of these narrative data is time-consuming, difficult to scale, and susceptible to interpretative bias [16-20]. Consequently, large volumes of narrative data collected with the MEDLO tool remain underanalyzed and underutilized for deriving QoL-related insights in dementia care.

However, recent advances in AI, particularly in natural language processing (NLP), hold at least a partial solution to the practical limitations of current methods for analyzing narrative data in long-term care. NLP focuses on the analysis and interpretation of human language in textual form by enabling automated computerized detection of themes, tones, and meaning within large volumes of narrative data [21]. In recent years, large language models (LLMs) have represented a significant advance in NLP, enabling models to interpret linguistic patterns with greater contextual and semantic depth than previous alternative approaches [22,23]. Trained on vast corpora of text, LLMs are capable of analyzing large volumes of text that would otherwise be impractical to analyze due to limited resources. LLM-based analysis can systematically extract recurring language patterns, emotional tone, and semantically related themes from large volumes of unstructured narrative data [24,25]. Therefore, LLMs could potentially also be used to identify QoL-relevant patterns in unstructured narrative data, with the potential to transform these data into interpretable QoL-related information [26]. Recent studies have used NLP and LLMs to extract information from free-text electronic health records [27-32]. Few studies have applied these methods within dementia care [27,33-35], and none have focused on QoL.

The study aims to explore whether LLM-based analyses can be used to structure existing narrative data, stemming from MEDLO data, into interpretable QoL-relevant patterns among people living with dementia residing in nursing homes in the Netherlands. Specifically, we examine whether N-gram analysis, sentiment analysis, and LLM-based topic modeling can identify recurring language patterns, emotional tone, and semantic clusters that can be mapped to Lawton QoL domains. As MEDLO was not developed as a QoL instrument, these outputs are interpreted as exploratory QoL-relevant signals rather than direct measurements of QoL. This study contributes to the field by showing how routinely collected observational text may be transformed into structured, QoL-relevant patterns for dementia care research

## Methods

### Study Design

This study builds on a previous exploratory observational study by analyzing the existing narrative data to explore whether LLM-based analyses can transform these narratives of daily life into interpretable insights of QoL in people living with dementia.

### Data Collection

The dataset used in this study was originally collected as part of the “Green Care Farms—an innovative care environment for adults living with dementia” project [36]. Similar to the current study, the Green Care Farms project was conducted within the Living Lab in Ageing and Long-Term Care in South Limburg, the Netherlands [37]. The Green Care Farms project aimed to monitor the daily lives of people living with dementia residing in Dutch care farms and traditional nursing homes, examining how the care farm environment influences their well-being.

Data were collected using the MEDLO tool, a tablet-based observation instrument specifically developed to capture detailed, real-time descriptions of the daily lives of people living with dementia in long-term care settings [14,15].

Data were collected over a period of 2 months per Green Care Farm, and the period of the day of the observation was selected randomly. Three researchers and 2 research assistants carried out observations at the 4 green care farms. All observers were trained by an experienced user of the MEDLO tool. This training consisted of studying the manual, practicing the training, and discussing the data. Within this training, both researchers observed the same people living with dementia and discussed their findings. The researchers conducted observations independently when they reached a complete agreement.

People living with dementia were observed for one morning (7 AM-11:30 AM), one afternoon (11:30 AM-4 PM), and one evening (4 PM-8:30 PM), with a 30-minute break each day. Every 20 minutes, a maximum of 11 people living with dementia were observed for 1 minute in a random sequence. This led to 12 observations per individual living with dementia per observation day and 36 momentary assessments per individual living with dementia in total. The people living with dementia were observed for 1 minute. By randomly selecting time points within the days of a person with dementia, insight into their daily lives was gained [15]. All observed people living with dementia had a formal diagnosis of dementia, confirmed by medical staff.

During each 1-minute observation, observers recorded both quantitative scores and narrative descriptions on four domains of daily living: (1) activity performance, (2) physical environment, (3) social interaction, and (4) emotional well-being. The narrative descriptions were written as free-text entries in natural language, without predefined categories or templates, allowing observers to capture contextual details and subtle nuances often overlooked in standardized formats [13,14]. For example (translated from Dutch): “She is sitting at the dining table next to the kitchen unit, staring at a newspaper in front of her.” These narrative descriptions served as the primary dataset for the current study’s NLP analysis.

### Ethical Considerations

Data were derived from the Green Care Farms study. This study was reviewed and approved by the Medical Ethics Committee of Zuyderland, the Netherlands (METCZ20210097). An amendment to the study protocol received a positive decision from the same ethics committee on February 20, 2024. The present study used anonymized observational data collected within this approved study protocol for secondary analysis. All procedures were conducted in accordance with the ethical standards of the institutional and/or national research committee and with the Declaration of Helsinki and its later amendments [38]. The data used in the present study had been anonymized by the researchers from the original study before being transferred to the current research team.

### Data Preprocessing

Preprocessing is a critical step in text mining, as it prepares raw text for analysis by removing noisy or unreliable data that may interfere with accurate interpretation [19,39]. In this context, “noise” refers to inconsistencies in the data that do not carry meaningful information for analysis [40]. For the current study, preprocessing and analysis were performed using Python version 3.11.8, a free software package for general-purpose programming language [41]. The code for all the analyses can be found on GitHub [42].

While traditional NLP pipelines often involve extensive text cleaning, such as tokenization, normalization, stop-word removal, stemming, and handling punctuation and numbers, minimal preprocessing was conducted in this study [43]. The rationale for this approach was to preserve the natural language, contextual detail, and subtle nuances in the narrative data [44]. These nuances, including emotional tone and subtle behavioral cues, are essential for understanding the QoL of people living with dementia.

Although this study applied minimal preprocessing, several preprocessing steps were carried out in collaboration with researchers from the original Green Care Farm project, leveraging their familiarity with the data to ensure high data quality. (1) All narrative data from the MEDLO tool were extracted from the Excel file into a plain text file and loaded into Python; (2) all words were lowercased to enable consistent matching of identical terms regardless of case and to facilitate abbreviation expansion; (3) commonly used abbreviations, introduced during data collection to expedite note-taking, were replaced with their full-length equivalents (eg, lr: living room, bk: big kitchen); (4) double spaces and stray blank spaces were removed, and numbers were written out in words to improve textual consistency; and (5) the final preprocessed text file was converted into a PDF format, which was then used as input for LLM-based analyses, allowing the model to process the narrative data in its original form. The conversion to PDF was a pragmatic workflow decision rather than an analytical preprocessing step. The secure analysis pipeline had originally been developed to process PDF input files, and therefore, the final preprocessed text file was converted to PDF to ensure compatibility with this existing pipeline.

### Model Architecture

This study analyzed the narrative data using an LLM approach, using GPT-4.0-mini deployed through the Azure OpenAI Service (Microsoft) [45]. The analysis was conducted in Python 3.11.8 using the OpenAI Python package (openai==1.59.6), which was used to send requests to the Azure OpenAI chat-completion end point [46,47]. Supporting packages included PyPDF2 for extracting text from the PDF input file and pandas for structuring the extracted text during analysis [48,49]. The extracted narrative text was combined into a single Dutch text corpus and submitted to the deployed model through the local Azure deployment named *4OMINI* using API version *2023-09-01-preview*. The request specified a maximum generated output length of 4096 tokens. Temperature, seed, and structured response format were not explicitly set in the request. Therefore, outputs were generated without explicitly controlling randomness or enforcing a structured response format [50-52]. The analyses were performed via the model between November 2024 and May 2025. The model was not trained or fine-tuned on these data but was used only for inference. All preprocessing, prompt preparation, and output storage were conducted within the local secure research environment of Maastricht University. This secure and scalable blueprint of the model enables organizations to use LLMs, such as GPT-4.0-mini, while safeguarding data privacy.

### Prompt Engineering

#### Overview

Prompt engineering was used to instruct the model in extracting language pattern insights from the narrative data. Three task-specific prompts were developed in Dutch: (1) N-gram identification and QoL-domain mapping, (2) sentiment scoring of N-grams, and (3) topic modeling with QoL-domain mapping. Each prompt specified the analytical task, the expected output format, the use of Lawton QoL domains as the conceptual framework, and instructions to remain close to the wording of the original observational narratives. The final prompts can be found at the repository at GitHub [42].

Prompt development followed an iterative refinement process [53]. After each analytical run, coauthors (DS, MCS, RFvdW, SA) evaluated whether the output was coherent, sufficiently specific, grounded in the narrative data, and consistent with the predefined QoL-domain framework. Prompt adjustments were made when outputs were too broad, semantically unclear, or inconsistent with the predefined QoL-domain framework. This iterative process continued until the coauthors reached consensus that the outputs were sufficiently interpretable, contextually plausible, and aligned with the study aim. In total, the N-gram identification and QoL-domain mapping prompt were run 9 times, the sentiment scoring prompt was run 6 times, and the topic modeling and QoL-domain mapping prompt were run 10 times. All prompt versions and corresponding outputs were stored to document the development process and are available from the corresponding author upon request. The final prompts were each executed once for the final analysis and were not repeated multiple times as identical runs to formally assess run-to-run stability. Therefore, some degree of output variability cannot be ruled out.

All analyses were conducted on the original Dutch narrative data. The narratives were not translated before analysis. English translations of selected examples are provided only for reporting purposes in this paper.

#### N-Gram Frequency Analysis and Sentiment Analysis

The first analysis performed by the model was N-gram frequency analysis, and the second analysis was sentiment analysis. To accomplish both analyses, 2 different prompts were developed to guide the model’s analyses: one prompt to identify the most frequent N-grams per QoL domain and the other prompt to determine the most positive and most negative sentiment-scoring N-grams per QoL domain.

An N-gram is a sequence of N consecutive words extracted from a text [54]. For example, the sentence “The resident smiled while listening to music” can be broken down into the following: (1) unigrams: “The,” “resident,” “smiled,” “while,” “listening,” “to,” “music;” (2) bigrams: “The resident,” ‘‘resident smiled,” “smiled while,” “while listening;” and (3) trigrams: “The resident smiled,” “resident smiled while,” “smiled while listening.” Using the first prompt, the model was instructed to identify the N-grams across the narrative data. The next step in this prompt was to categorize each N-gram into an appropriate QoL domain. The mapping of N-grams to QoL domains was operationalized using the Lawton QoL framework. The definitions of behavioral competence, psychological well-being, objective environment, and perceived QoL were added to the context section of the prompts. Based on these definitions, the model was instructed to assign each N-gram to the most appropriate QoL domain. Consequently, the prompt instructed the model to report the 15 most frequent N-grams per QoL domain. The 15 N-grams assigned to each QoL domain were then reviewed by the coauthors and cross-referenced with the literature to assess whether they were contextually plausible and consistent with the domain to which they had been assigned.

In cases where an N-gram appeared too ambiguous to convey a clear meaning, the model was instructed, using the first prompt, to extend the N-gram by one additional word, derived from the original narrative context, rather than generated independently, in order to improve the clarity of the N-gram. For instance, the trigram “is happy with” could be extended to “is happy with visits” depending on its original context. This approach could ensure that N-grams reflect meaningful language units rather than isolated, ambiguous terms. Consequently, using the second prompt, the model identified the most positive and most negative sentiment-scoring N-grams categorized per QoL domain. Specifically, this prompt first identified all N-grams present in the narrative data and then applied sentiment analysis to all N-grams. Thus, sentiment analysis was not performed on the most frequent N-grams, from the first prompt, but on all N-grams identified in the narrative data. N-gram sentiment analysis involves determining the emotional tone of a given text [19,39,55]. For example, “The resident smiled while listening to music” would typically be classified as positive, whereas “The resident sat alone and looked down without responding” would be classified as negative. Neutral sentiment could be applied to emotionally neutral statements such as “The resident walked across the room.”

Each identified N-gram was assigned a sentiment score on a scale from −1 (“very negative”) to 1 or ambiguous sentences (“very positive”), with 0 indicating neutral sentiment. The model was instructed to apply both lexicon-based and context-aware sentiment score approaches to improve the accuracy of the sentiment scores assigned to the N-grams. Lexicon-based sentiment analysis relies on predefined emotional associations of individual words; for instance, “happy” is typically associated with positive sentiment. However, this method may misclassify more complex or ambiguous sentences. To address this complexity, a context-aware sentiment analysis was also used to interpret the meaning based on phrasing and tone. For example, although the sentence “The resident smiled briefly at the caregiver but avoided eye contact and stayed silent” included the word “smiled,” the overall sentiment might be neutral or negative due to the surrounding disengagement indicators.

While the most positive and most negative sentiment-scoring N-grams of each QoL domain were identified based on their sentiment scores, it should be noted that these N-grams might have appeared only once in the narrative data. As such, these most positive and most negative sentiment-scoring N-grams do not necessarily provide insight into a general sentiment tendency within each QoL domain. To gain insight into broader sentiment patterns, weighted sentiment summaries were calculated [56]. For each N-gram of the most positive and most negative sentiment-scoring N-grams in a QoL domain, the sentiment score of that N-gram was multiplied by its frequency of occurrence in the narrative data. Subsequently, the sum of these products was then divided by the total frequency of all the N-grams within each QoL domain. This yielded a weighted sentiment score for each QoL domain.

#### Topic Modeling With QoL Domain Mapping

The third analysis regarded topic modeling, which was used to identify recurring themes in the narrative data. This analytical task was instructed via a third prompt. Topic modeling refers to the process of grouping semantically related keywords into overarching themes [19,57,58]. For example, keywords such as *pacing, fidgeting, frustration, wandering,* and *repetitive movement* might be clustered under the topic label *agitation and restlessness*. In this study, topic modeling was implemented as an LLM-based analytical task rather than as a traditional statistical topic modeling [59,60]. The definitions of Lawton 4 QoL domains were included in the context section of the prompt. The model was instructed to group semantically related words into topics, assign a descriptive label to each topic, and then link each topic to one or more QoL domains (as a single topic might reflect more than one aspect of QoL) based on the associated words in the narrative data [60,61]. Coauthors then reviewed whether the topics with included keywords, their labels, and their assigned QoL domains were coherent, plausible, and consistent with the Lawton framework.

The prompt also included guidance to enhance analytical granularity, such as splitting broad topic clusters into more specific clusters. To improve the accuracy of the topic modeling analysis over time, the model was further guided, via the prompt, to suggest refinements to the prompt itself. As with the N-gram and sentiment analysis, prompt development followed an iterative process, with adjustments to structure, wording, and level of detail made in response to the relevance and coherence of earlier outputs.

### Expert Model Evaluation

Expert evaluation was used to assess whether selected model outputs were meaningful and contextually plausible in relation to long-term care practice. For sentiment analysis, 2 researchers (LF and KR, both with 5 years of research experience in long-term care) from the original Green Care Farm project independently reviewed the top positive and negative N-grams per QoL domain [62]. They assessed whether the assigned sentiment direction and intensity were plausible, given the wording of the N-gram and its interpretation within the context of observational dementia care. Disagreements between reviewers were recorded and discussed, and the final interpretation was based on consensus.

For topic modeling, the model-generated clusters were reviewed by 4 members of the research team (DS, HC, SA, and MCS), with expertise in dementia care and QoL research, long-term care research, and health care AI methods. The topic clusters were assessed using 2 qualitative criteria: coherence and distinctiveness. Coherence referred to the internal consistency of each topic cluster, that is, whether the terms grouped under a given label were meaningfully related [58]. For example, a cluster containing the terms *music, dancing, laughter, and clapping* was assessed to be coherent and appropriately labeled as *leisure activities*. In contrast, if an unrelated term, such as *agitation,* appeared within that same cluster, it indicated a lack of coherence, which resulted in further fine-tuning of the prompt. Distinctiveness was assessed by examining the boundaries between topic clusters to avoid redundancy or overlap. If an overlap between topic clusters was identified by the research team, the model was iteratively prompted to generate more distinct clusters. This process of prompt engineering continued until the research team reached consensus that the topic clusters were sufficiently distinct, with minimal thematic overlap.

## Results

### Overview

In total, 5652 observations were conducted on 151 people living with dementia during the Green Care Farms study [62]. Narrative descriptions were missing in 30 (0.53%) observations. The narrative descriptions totaled 50,106 words. An average of 10.5 (SD 5.8) words was noted per narrative. Table 1 presents the background characteristics of the 151 individuals living with dementia who were observed.

**Table 1.** Characteristics of people living with dementia observed in the Green Care Farm study.

Characteristics	Participants (N=151)
Age, mean (SD)	84.7 (7.1)
Sex (female), n (%)	108 (72)
S-MMSE^a^ score, mean (SD)	10.5 (7.4)
Barthel Index score^b^, mean (SD)	13 (5.6)

a S-MMSE: Standardized Mini-Mental State Examination. The S-MMSE assesses global cognitive functioning, with lower scores indicating greater cognitive impairment.

b The Barthel Index measures independence in activities of daily living, with lower scores indicating greater dependency. The table is adapted from the original study [62].

### N-Gram Frequency Analysis

The N-gram frequency analysis revealed language patterns related to QoL. Frequently observed terms included *laughing, smiling, satisfied*, and *nice,* suggesting that people living with dementia were often described as being in a state of positive emotional well-being. Negative affective expressions, such as *becoming angry, crying*, and *feeling distressed*, appeared far less frequently than positive affective expressions.

Ten of the 15 N-grams within the QoL domain behavior competence reflected low-activity behavior. Observers frequently noted people living with dementia *sitting at the table, reading the newspaper*, or *looking outside*. Although terms that encompass “activity” such as *helps with cooking* or *participates in gymnastics* were present, they were less common. This suggests a pattern of predominantly passive engagement in daily life, despite generally positive emotional expressions.

The daily lives of people living with dementia were most often observed in 2 core settings: *the private room* and *the living room*. Additionally, a few observations of people living with dementia were made in outdoor spaces, such as a *terrace*, *the garden*, or *in the sun*.

**Figure 1.**
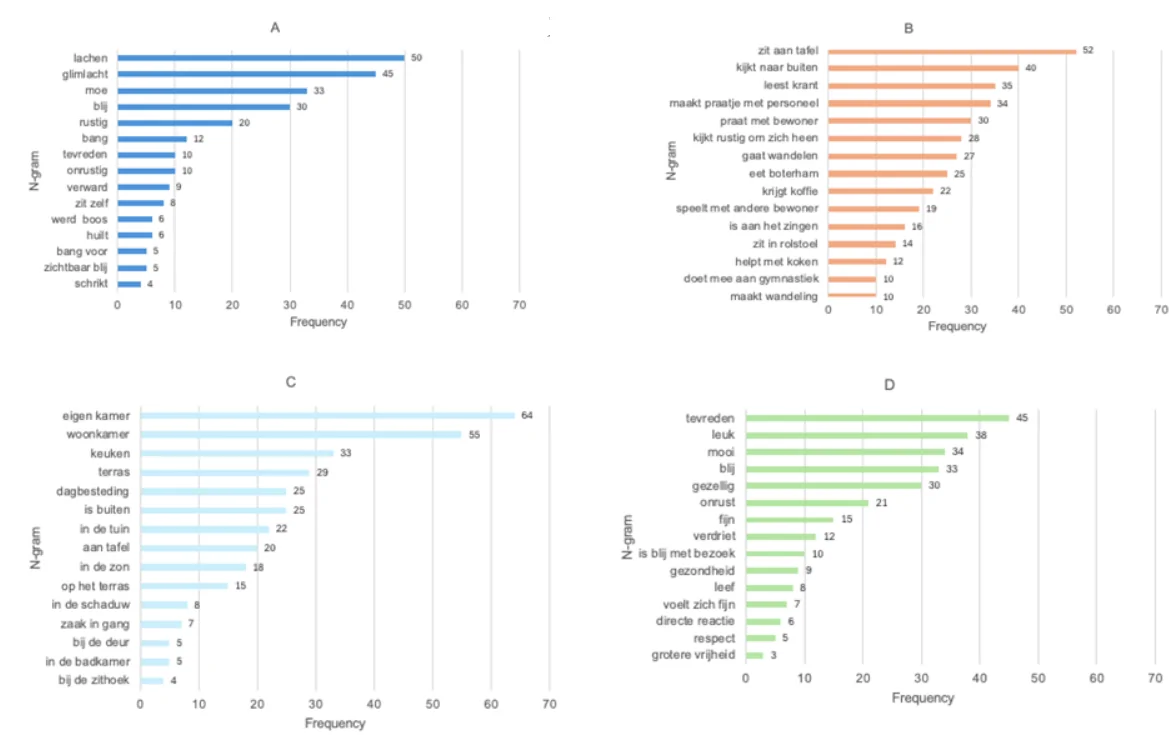
Most frequent N-grams in observational field notes, categorized per the Lawton quality of life (QoL) domain. (A) N-gram frequency psychological well-being domain; (B) N-gram frequency behavioral competence domain; (C) N-gram frequency objective environment domain; (D) N-gram frequency perceived QoL domain.

Similar to other QoL domains, N-grams within the perceived QoL domain were mainly positive affective expressions, such as *satisfied, nice, beautiful*, and *happy*. The partial overlap in N-grams from the domain of psychological well-being was observed (ie, *satisfied* and *happy*). The 15 most frequent N-grams identified within each QoL domain are presented in Figure 1.

Figure 1 shows the 15 most frequent N-grams identified in the observational field notes for each of Lawton QoL domains. Frequencies indicate the number of times each N-gram occurred in the contextualized text segments included in the N-gram analysis. The panels show that psychological well-being was mainly represented by affective expressions such as “lachen,” “glimlacht,” and “blij;” behavioral competence by passive activity phrases such as “zit aan tafel” and “kijkt naar buiten;” objective environment by indoor settings such as “eigen kamer” and “woonkamer;” and perceived QoL by evaluative or experiential expressions such as “tevreden,” “leuk,” and “mooi.”

Although the model was instructed to extend ambiguous N-grams by one word to improve clarity, some N-grams still lacked semantic clarity and contextual relevance. For instance, certain N-grams *live* or *direct reaction* offered limited insight.

### Sentiment Analysis

**Figure 2.**
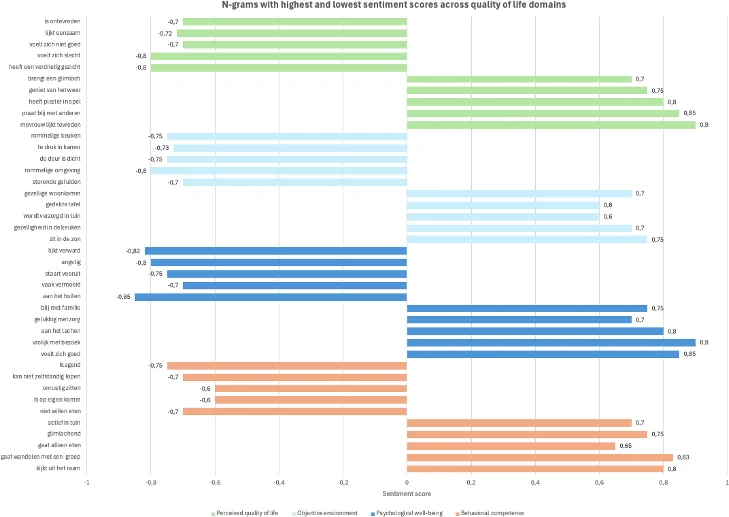
Sentiment scores of positive and negative N-grams by Lawton quality-of-life (QoL) domains.

Figure 2 presents the 5 most positively and negatively scored N-grams within each QoL domain, showing a wide range from strongly negative to strongly positive sentiment. The analysis of the N-grams identifies distinct sentiment patterns across QoL domains. High positive sentiment-scoring N-grams (around +0.8 to+0.9) show engagement and enjoyment of people living with dementia, such as *experiencing fun during games*, *being cheerful with visitors*, and *going walking with a group*. On the contrary, strongly negative sentiment-scoring N-grams (around −0.8) appear in expressions such as *crying*, *appearing confused,* and *feeling anxious*, which predominantly capture emotional distress in people living with dementia.

Within each QoL domain, the sentiment polarity of the N-grams is clear. Perceived QoL ranges from *female client seems satisfied* to *feels bad*. Psychological well-being ranges from *happy with visitors* to *crying*. Behavioral competence ranges from *goes walking with a group* to *complaining*.

While these examples reflect a mix of positive and negative-scoring N-grams, the overall sentiment distribution in the narrative data was skewed. Across all QoL domains, negative sentiment outweighed positive sentiment. Positive sentiment made up between 17.9% and 24.9% of the total weighted sentiment, depending on the QoL domain. This suggests that the average sentiment per QoL domain was driven downward by the higher intensity of negative sentiment-scoring N-grams. Even when positive sentiment-scoring N-grams appeared more frequently, their lower sentiment scores were insufficient to offset the impact of fewer but more intensely negative sentiment-scoring N-grams. Table 2 presents the weighted positive and negative sentiment scores for each QoL domain.

Figure 2 shows sentiment scores for the top 5 most positive and negative N-grams categorized by Lawton QoL domains. Bars to the right of 0 represent positively scored N-grams, whereas bars to the left of 0 represent negatively scored N-grams. Colors indicate the QoL domain to which each N-gram was assigned: behavioral competence, psychological well-being, objective environment, or perceived QoL. These sentiment scores reflect the polarity of documented textual expressions and should not be interpreted as direct measures of participants’ QoL.

**Table 2.** Weighted sentiment scores for the most positive and negative N-grams by Lawton quality-of-life (QoL) domains.

QoL domain	Positive (wsum^a^)	Negative (wsum)	Pos/neg ratio^b^	%Positive^c^	%Negative
Behavioral competence	43.1	140.0	0.3	24.9	75.1
Psychological well-being	39.9	182.5	0.2	17.9	82.1
Objective environment	38.9	153.7	0.25	20.2	79.8
Perceived QoL	42.8	135.0	0.3	24.4	75.6

a wsum: weighted sum of sentiment scores, calculated as N-gram frequency multiplied by the sentiment score.

b Pos/neg ratio: ratio of positive to negative weighted sentiment.

c Percentage weighted sentiment: proportion of positive or negative sentiment relative to the total weighted sentiment per QoL domain. These scores reflect sentiment patterns in documented observational text and should not be interpreted as direct measures of participants’ QoL.

### Topic Modeling

Topic modeling identified 8 main topics in the narrative data. Each topic was defined by a set of representative keywords and was linked to one or more QoL domains, as shown in Table 3.

The model inferred thematic correlations based on the co-occurrence of keywords across different QoL domains. This showed that narrative data involving social interaction and daily activities (ie, *eating* and *walking*) frequently appeared alongside positive emotional expressions (ie, *smiling* and *feeling relaxed*). On the contrary, negative emotional states (ie, *restlessness* or *sadness*) were often reported in contexts of social isolation or reduced stimulation, such as when people living with dementia remained in bed or stayed alone in their rooms.

Perceived QoL was linked to the highest number of topics (6/8). The other QoL domains appeared in 4 topics. Many topics were assigned to multiple QoL domains, indicating that a single thematic cluster often reflects multiple QoL domains. For example, the topic *eating and drinking* was linked to behavioral competence, objective environment, and perceived QoL, reflecting the multidimensional nature of QoL.

**Table 3.** Overview of identified topics including keywords and associated Lawton quality-of-life (QoL) domains.

Topic^a^	Keywords	QoL domains
Daily activities	Sleeping, eating, singing, reading, and cooking	Behavioral competence, objective environment, and perceived QoL
Psychological well-being	Laughing, smiling, angry, confused, and content	Psychological well-being and perceived QoL
Communication with staff and coresidents	Talking, conversations, and singing	Psychological well-being and perceived QoL
Physical activity and mobility	Walking, grocery shopping, walking around, and moving	Behavioral competence and objective environment
Personal care and support	Dressing, showering, medication, and helping	Behavioral competence and psychological well-being
Social interaction	Visits, family, residents, and being together	Psychological well-being and perceived QoL
Eating and drinking	Sandwich, coffee, soup, tea, cookie, and meal	Behavioral competence, objective environment, and perceived QoL
Environmental factors	Terrace, garden, kitchen, living room, sun, and flowers	Objective environment and perceived QoL

a Topics were identified from the observational field notes and linked to Lawton QoL domains based on the content of the associated keywords.

## Discussion

### Principal Findings

The study aimed to explore whether an LLM could be used to structure existing MEDLO narrative data into interpretable QoL-relevant patterns in people living with dementia residing in nursing homes in the Netherlands. Specifically, it examined whether N-gram analysis, sentiment analysis, and LLM-based topic modeling could identify recurring language patterns, emotional tone, and semantic clusters that could be mapped to the Lawton QoL domains.

A first implication from this study concerns the need to incorporate context into observational data when reporting and analyzing affect in people living with dementia. The findings from this study indicated that while people living with dementia were predominantly observed during passive activities and in a limited range of indoor settings, they were often observed expressing positive emotions during the day. In addition, the sentiment analysis showed that although negative expressions were documented less frequently, they received more extreme negative sentiment scores. This finding is consistent with the literature, suggesting that people living with dementia are frequently documented as comfortable and content in their daily lives; however, their well-being remains sensitive to moments when physical, emotional, or social needs are not fully met [3,63-65]. The reported high-intensity negative expressions may reflect moments when these needs are not adequately addressed [66,67]. However, measuring emotions in people living with dementia is challenging because their emotional expression is often characterized by neutral affect [63]. Consequently, in this study, observations reflecting neutral affect may have contributed little to the weighted sentiment scores, while less common negative expressions could have stood out against this neutral baseline and received relatively high-intensity scores, thereby influencing the weighted sentiment results.

Hence, there is a need to develop documentation guidelines to encourage observers to standardize their data collection methods and practices. While traditional NLP methods, such as term frequency-inverse document frequency, rely on word occurrences, LLMs interpret meaning from the surrounding context [23]. Consequently, the descriptive detail of the narrative data directly affects the outputs. This dependency on context-rich narrative data means that variation in how observers phrase their descriptions can lead to systematic differences in model outputs, even when experiences are similar [67-69]. For example, what to record, how detailed to be, and which emotions or behaviors to emphasize are each influenced by time constraints and professional judgment. Therefore, developing documentation guidelines could encourage observers to standardize their documentation. An example of such documentation guidelines is to include narrative information about the social context, setting, and activity, alongside emotional expressions, in care descriptions, which could provide richer input for LLM-based models while remaining feasible within care routines [70-72]. This also aligns with broader debates in health care–based LLM use, where the quality and style of input text have been shown to shape model performance in long-term care, potentially improving care for people living with dementia [70-72].

A second implication of this study concerns the ways in which LLM-generated conceptualizations of QoL can provide insight into how different aspects of QoL can co-occur and interact in dementia care and in what circumstances they do so [62,66,73-75]. The Kitwood personhood framework, along with later work on person-centered care, emphasizes that QoL derives from opportunities for purposeful activity, social reciprocity, and recognition of individuality rather than from momentary affective expressions alone [67,76,77]. From this perspective, positive documented expressions during passive activities in low-variation indoor settings may be interpreted as reflecting momentary positive affect, while leaving unanswered questions about the extent of meaningful engagement [78,79]. In our analysis, the LLM-generated outputs linked affective terms with descriptions of activity, agency, and social interaction. These textual patterns may provide contextual information that could support further exploration of the distinction between momentary positive affect and meaningful engagement. Furthermore, the topic modeling in this study provides support for the idea that all of Lawton QoL domains are interwoven [3,6,80]. For example, the description of *enjoying helping with cooking* may simultaneously reflect behavioral competence, psychological well-being, and perceived QoL. In the future, LLM-generated clusters may help describe how emotional, physical, social, and environmental aspects co-occur within observational narratives, while existing questionnaires currently isolate experiences into predefined categories [81-83].

In sum, this study aligns with broader developments in health care, where increasing attention is being given to the analysis of narrative data [24,84-87]. The current findings demonstrate that even brief narrative care data can be structured into recurring textual patterns. This creates opportunities to integrate near–real-time feedback loops into tools such as the MEDLO. Embedding LLM-based analysis within such tools may enable the immediate analysis of narrative descriptions, transforming routine documentation into dynamic QoL feedback of people living with dementia. For example, the automatic detection of subtle changes in emotional tone or activity diversity could help care teams recognize early signs of reduced well-being. Such insights may enable staff to identify emerging needs earlier and adjust activities, environments, or interactions in a more proactive and person-centered manner [67,88,89]. Importantly, these integrated feedback loops should complement, not replace, the expertise of care professionals by offering timely, data-informed insights to support daily practice [90,91]. Evidence from long-term care indicates that these technologies must be developed in close collaboration with domain experts, including care professionals, researchers, and technology developers, to ensure integration into daily workflows, meet usability requirements, and uphold ethical standards [92-95].

### Strengths and Limitations

A key strength of this study was the unique nature of this real-world dementia-specific dataset. The manual analysis of this dataset would be very time-consuming. However, analyzing these data with an LLM enabled the systematic exploration of thousands of brief descriptions. Another strength lies in the decision to use minimal preprocessing, preserving the authenticity and contextual richness of the observational language. This approach enabled the LLM to capture the nuances of natural expression, thereby maintaining practical relevance in real-world care contexts. Transparency and reproducibility were also prioritized: all coding materials and prompts were made openly available, supporting replication and further adaptation of the analytical workflow in other settings. Finally, the black-box nature of LLMs limits insight into how specific outputs were generated. To mitigate this, 2 experts manually reviewed the most positive and most negative-scoring N-grams to assess whether the assigned sentiment scores aligned with their contextual meaning.

Several limitations should also be acknowledged. First, individual narrative descriptions were often brief and fragmented with an average length of 10.5 (SD 5.80) words. This limited the semantic richness available for LLM-based analysis and may have affected the quality of all outputs. However, analyzing the entire corpus at scale partly offset this limitation, allowing recurring patterns to emerge, despite the brevity of individual notes. Second, the data were collected and analyzed in Dutch nursing home settings, which may limit the generalizability and transferability of the findings to other long-term care contexts and languages. However, analyzing the narratives in their original language avoided potential loss of meaning that could have occurred through translation before analysis. Third, although the final prompts were developed through an iterative refinement process, each final prompt was executed only once for the final analysis. LLM-based outputs may vary across repeated executions and model versions, even when prompts and input data remain unchanged [50,96]. We documented the model, deployment, API version, software package version, analysis period, prompts, and code; however, exact computational reproducibility cannot be guaranteed. Fourth, although the expert panel evaluated the sentiment scores of the top-scoring N-grams, the analysis relied on a general-language lexicon rather than a dementia-specific lexicon. Consequently, some context-dependent terms may have been mis-scored and, if they did not appear among the reviewed N-grams, remained underutilized. Finally, the expert review assessed whether selected outputs were coherent, plausible, and meaningful in relation to long-term care practice but did not perform formal validation or allow for the calculation of agreement metrics. Because QoL in dementia is subjective and lacks a single objective gold standard for validation, the findings should be interpreted as exploratory QoL-relevant patterns rather than validated classifications or measurements of QoL [66].

### Future Research

Future research should build on these findings in several directions. First, larger and more comprehensive datasets, including greater and richer volumes of observational narrative descriptions, are needed to strengthen the robustness and generalizability of the analysis. Second, a dementia-specific lexicon should be developed and validated to ensure that terms are weighted appropriately in this context. Third, prospective validation studies are required to examine whether LLM-based themes and sentiment patterns correspond to established QoL instrument scores. Fourth, research should explore the real-time integration of LLM-based analysis into observation tools such as MEDLO, evaluating usability, interpretability, and workflow alignment through co-design with care professionals.

These findings should be interpreted as exploratory. Although the current analysis suggests that LLM-based analyses may help structure observational narratives into QoL-relevant patterns, the approach is not yet validated against QoL measures. Before such outputs can inform person-centered care, further research is needed to examine whether LLM-generated outputs correspond to established QoL measures if validated and integrated into care workflows.

### Conclusion

LLM-based analyses predominantly identified passive activities in little variation in indoor settings; however, people living with dementia were often described as having a positive affect. These findings suggest that LLM-based analyses can structure observational narratives into interpretable patterns of activity, emotional expression, and social interaction. Further validation is required before such outputs can support multidisciplinary reflection and person-centered dementia care.

## Abbreviations

LLM: large language model
MEDLO: Maastricht Electronic Daily Life Observation
NLP: natural language processing
QoL: quality of life
